# Access to general practice in England: political, theoretical, and empirical considerations

**DOI:** 10.3399/bjgp16X686977

**Published:** 2016-08-26

**Authors:** Thomas E Cowling, Elinor J Gunning

**Affiliations:** National Institute for Health Research Doctoral Research Fellow, Department of Primary Care and Public Health, Imperial College London, London.; Department of Primary Care and Public Health, Imperial College London, London.

Access to general practice services in England has been a prominent theme in recent issues of the *BJGP*. Simpson and colleagues^1^ outlined the historical context of current policy to extend practice opening hours in the evenings and at weekends. Campbell and Salisbury^2^ examined the conceptual foundations of access to health care. Ford and colleagues^3^ reported empirical work on patient preferences for additional opening hours, while Scantlebury and colleagues^4^ modelled general-practice-level determinants of emergency department visits. We extend this discussion below, focusing on the UK government’s controversial commitment for all patients in England to be offered GP appointments between 8 am and 8 pm, 7 days a week, by 2020.^5^

## POLITICAL CONSIDERATIONS

Language used by the government when referring to its commitment to extend opening hours, in addition to that used for its wider political strategy, provides one means of analysing this policy. Relevant government press releases often refer to people with busy work and family lives who struggle to fit in GP appointments; the latest mentioned *‘7-day GP services for hardworking families’* and offering *‘hardworking taxpayers and families the security of care they need’* .^6^ In April 2015, at the launch of the Conservative Party manifesto for the last UK general election, David Cameron declared the Conservatives to be *‘the party of working people’*.^7^ In October 2015, after being re-elected as Prime Minister, he repeated this position at the Conservative Party conference: *‘The party of working people, the party for working people — today, tomorrow, always.’* 8 The consistent rhetoric, highlighting a focus on the employed, is one sign that the policy to extend opening hours cannot be divorced from wider political activity.

The timing, source, and place of the government’s statements on this policy issue are also revealing. The Prime Minister, rather than the Department of Health or NHS England, has often made the major relevant announcements. These have taken place, for example, at the Conservative Party annual conferences in September/October 2013, 2014, and 2015. The first commitment in the Conservative Party election manifesto read, *‘We will continue to increase spending on the NHS, provide 7-day a week access to your GP and deliver a truly 7-day NHS.’* 9 This highlights that the policy to extend opening hours is seen as a politically important issue — likely to win election votes and in keeping with the Conservative Party strategy to position itself as the party that most benefits working people. Such policy could face organised medical opposition, however, as with recent strikes against changes to junior doctors’ contracts also linked to the ‘7-day NHS’ agenda.

Government plans for general practice do not appear likely to change soon. When asked in parliament about the aim of 7-day working, the Secretary of State for Health replied, *‘Increasing convenience for the general public in terms of being able to make routine evening and weekend appointments is a manifesto commitment that this Government made, so we have to honour that.’*^10^ A strong political element to this policy is clear.

What is less clear is how extended opening hours came to dominate policy direction on improving general practice services. Other interventions, such as telephone and online video consultations and increased use of healthcare professionals other than GPs, have been piloted alongside extended opening hours nationally as part of the Prime Minister’s GP Access Fund. It may be partly because opening hours are easily quantified, monitored, and communicated to the public in policy announcements; a ‘truly 7-day NHS’ including general practice has face value with voters. One concern is that opening hours have been conflated with access itself by many policymakers, without valid theoretical reason.

## THEORETICAL CONSIDERATIONS

The traditional account of definitions in philosophy literature states that the meaning of a term in a proposition is revealed by the empirical observations needed to verify the proposition as true or false.^11^ We cannot tell simply from observing a given general practice’s opening times whether a patient was ‘able to access care’ in that practice on their last attempt. Equally, we cannot infer the practice’s opening times solely from the proposition that the patient was (or was not) ‘able to access care’ on that attempt. The meaning of the term ‘access’ in this context is distinct from variables regarding opening times.

We can, however, infer whether a patient was able to access care on a given attempt by observing whether they then received care from their general practice. This reveals how we understand ‘access’ in common language and therefore its meaning. Opening times are better seen as a practical determinant of the probability that a patient is able to access care on a specific attempt, and a determinant of when care can be received. Because access and opening hours are theoretically distinct, their true relationship must be determined empirically.

## EMPIRICAL CONSIDERATIONS

The national evaluation of the first GP Access Fund pilot schemes did not validly test their impact on patients’ access to care or their attitudes towards opening times, despite these being key outcome measures for the schemes.^12^ In general, the evaluation was limited by poor data quality and the absence of rigorous methods designed to estimate the interventions’ causal effects. Caution should therefore be taken over some claims made by the evaluation, such as a 15% reduction in certain types of emergency department visits. Any effect estimate is unlikely to represent the effect of implementing the interventions nationally, because the pilot schemes are a self-selected group that may stand to benefit the most. Many interventions have been trialled simultaneously or introduced progressively, so the independent effects of extended opening hours are also difficult to estimate. The evaluation reported that medium-sized pilots provided, on average, around 41 minutes of extended hours per week per 1000 patients.^12^ This is not a large change to opening hours and the scope for some benefits would therefore seem limited.

The government has used several rationales to justify its policy to extend opening hours, so it is unclear what the main expectations are. One line of reasoning is that *‘... public satisfaction with access to GPs is falling. People are simply finding it too hard to see their GP’*, particularly working people.^13^ Data from the GP Patient Survey lend some support to these claims. Several measures relevant to appointment convenience, overall experience, and satisfaction with opening hours have decreased in recent years.^5^ Still, Table 1 shows that most people (79.7%) in England find their general practice’s opening times convenient.^14^ Those unable to take time off work to see a GP are much less likely to find current times convenient (55.8%), but they only account for around 18.7% of the population. The most frequent category of patients who find current times inconvenient can take time off work to see a GP (41.9% of ‘inconvenient’ responses). Extended opening hours could benefit both of these groups, yet little evidence addressing this hypothesis is available.

**Table 1.** Responses to the question ‘Is your GP surgery currently open at times that are convenient for you?’ in the GP Patient Survey 2013–2014, by employment category

**Employment category**	**Question response, *n* %**		**Total**

	**No (inconvenient)**	**Yes (convenient)**	
Not working^b^	28 936 (8.6)	308 818 (91.4)	337 753
Can take time off work to see GP	66 213 (22.3)	231 050 (77.7)	297 263
Cannot take time off work to see GP	62 911 (44.2)	79 504 (55.8)	142 415

Total	158 059 (20.3)	619 371 (79.7)	777 430

a Data were missing for 7.9% of responses; responses of ‘Don’t know’ are excluded from the table (6.6% of weighted responses).

b Full-time education, unemployed, sick or disabled, retired, looking after home, other. Responses are weighted to account for survey design and non-response (by age, sex, geographical location, general practice, and other variables) to increase national representativeness.^14^

One unanswered question is the amount by which opening hours should be extended, and when, to achieve the expected benefits for patients. The GP Patient Survey^3^ and the national pilot scheme evaluation^12^ both suggest that demand for GP appointments on Sundays is often likely to be low, at least in the short term. This finding challenges the government’s commitment for all patients to be offered GP appointments 7 days a week. NHS England’s response will help reveal the balance of political factors and empirical evidence on this issue.

## ANOTHER FRAME

This article has concentrated on government plans for general practice in terms of access and opening hours. This is often the frame used in relevant announcements,^6^ but there is a wider programme of change occurring. For example, the GP Access Fund has not only supported practices to trial new interventions; pilot schemes have also established new structural arrangements with greater collaboration between providers to offer additional services to larger populations.^12^ Clinical Commissioning Groups are also taking on new responsibilities for commissioning general practice services. The NHS Five Year Forward View outlined several new models of organising the NHS, some particularly radical such as vertically integrated ‘Primary and Acute Care Systems’ that are accountable for all care provided for a population under a capitated budget.^15^ From this perspective, extending general practice opening hours is just one intervention among wider change. It is, however, an intervention that the public can immediately grasp and intuitively favour. As such, it is now also a manifesto commitment for the Secretary of State to deliver.
